# The impacts of multiple obesity-related interventions on quality of life in children and adolescents: a randomized controlled trial

**DOI:** 10.1186/s12955-020-01459-0

**Published:** 2020-07-06

**Authors:** Hua Diao, Hong Wang, Lianjian Yang, Ting Li

**Affiliations:** School of Public Health and Management, Chongqing Medical University, Research Center for Medicine and Social Development, Collaborative Innovation Center of Social Risks Governance in Health, Chongqing Medical University, Chongqing, China

**Keywords:** Obesity, Overweight, Quality of life, Physical exercise, Diet control, Health education

## Abstract

**Background and purpose:**

Obesity has become a serious public health problem and family- and school-based interventions including physical exercise and diet control have been widely applied to attempt to combat this issue. The purpose of our study was to verify the effectiveness of an obesity-related comprehensive intervention model aimed at improving quality of life (QoL) among adolescents.

**Methods:**

A cluster randomized controlled trial (RCT) was conducted involving 948 subjects who were divided into an intervention group (*n* = 518) and a control group (*n* = 430). The intervention group received 1 year of obesity-related health education, physical exercise, and diet control. Their baseline body mass index (BMI) was calculated, and their QoL and basic information were assessed both before and after the intervention period using a self-designed Adolescent Quality of Life Scale and a basic information questionnaire.

**Results:**

After the intervention, significant differences in the psychological, social, and pubertal dimensions, and in total QoL (*P* < 0.05) were observed in the intervention group relative to the control group. Improved psychological QoL in the intervention group was our most robust study finding, with increases in psychological (B = 1.883, SE = 0.646, *P* = 0.004), pubertal (B = 0.853, SE = 0.296, *P* = 0.004) and total (B = 3.024, SE = 1.214, *P* = 0.013) QoL all being higher in this group. This intervention effect was found to be more substantial in boys than in girls.

**Conclusions:**

Family-individual-school-based interventions combining obesity-related health education, physical exercise, and diet control can improve psychological, pubertal, and total QoL in children, with these effects being most pronounced in boys.

**Trial registration:**

retrospectively registered NCT02343588.

## Introduction

Rates of obesity are rapidly increasing throughout the world, posing a serious public health concern. Globally, the prevalence of obesity is highest in developed countries, while two-thirds of obese individuals hail from developing countries. Between 1975 and 2016, the percentage of obese adults increased almost threefold [1]. Moreover, the average weight of children had risen over 5 kg within the last 30 years in the United States, with even more rapid increases in low- and middle-income countries between 2002 and 2018 [2–4]. In China, approximately 43% of adults and 20% of children are overweight or obese owing to changes in traditional lifestyle such as the increased popularity of Western fast food, declines in the regular practice of physical activity, and increased prevalence of a sedentary lifestyle [5]. It is therefore important that more efforts be made to combat this obesity problem.

Being overweight or obese is extremely harmful and can affect both physical and psychological health, increasing the risk of chronic non-communicable diseases including type 2 diabetes, hypertension, and fatty liver disease [6, 7]. In recent years, a strong link between obesity and various cancers has also been identified [8]. Obesity is thought to contribute to approximately 16–20% of cancer-related deaths in women and 14% in men [9]. From a psychological perspective, being overweight or obese increases the susceptibility of children to depression, anxiety, emotional disorders, and mood disorders [10, 11]. Horae et al. [12] found that children who are overweight or obese are 1.83 times more likely to suffer from depression than their normal-weight counterparts. Similarly, Sanderson et al. [13] found that children who were overweight or obese were more likely to suffer from mood disorders over the course of a 20-year longitudinal cohort study. In contrast, maintaining a normal body mass index (BMI≦25 kg/m^2^) is a protective factor with the potential to prevent up to 90,000 cancer deaths per year in the United States alone [14]. It is thus very clear that being overweight or obese can adversely affect quality of life (QoL) in children.

The exact genetic and environmental factors that influence obesity are complex [15]. Genes are thought to be major contributors to the development of these metabolic conditions, while environmental factors such as diet and exercise are generally regarded as being substantially easier to change in order to prevent excess weight gain [16, 17]. Lifestyle modifications including dietary changes and physical activities remain the foundation for optimal prevention and treatment strategies in overweight and obese children in existing studies [18, 19]. In combination with exercise, diet can further improve levels of high-density lipoprotein (HDL) cholesterol, fasting glucose, fasting insulin, and psychological health [7, 20]. Despite these clear advantages, most individuals have substantial difficulties maintaining dietary- and exercise-based interventions over extended time periods [17]. In general, after acute intervention efforts end, most subjects revert to their original lifestyle. Therefore, it is important that a health education approach be employed so as to improve the positive attitudes and behaviors of these individuals.

In summary, being overweight or obese can adversely impact the QoL of children and adolescents, and obesity-related interventions can improve QoL in obese children. However, children often have difficulty maintaining the behaviors introduced during these intervention efforts. Therefore, the aim of present study was to evaluate the impact of a comprehensive family-individual-school-based intervention approach involving obesity-related health education, diet control, and physical exercise on the QoL of Chinese children and adolescents.

## Methods

### Study design, participants, and process

A cluster-based randomized controlled trial (RCT) involving 4 schools was conducted in ShaPingBa district, ChongQing. Two primary schools and two middle schools were randomly selected from this district. Next, four to six classes were chosen randomly from grades 4–6 in these primary schools and grades 7–9 in these middle schools. Primary schools and middle schools were each randomly separated into 2 groups: an intervention group that received comprehensive obesity-related interventions and a control group that received no specific interventions. Based on previous similar studies and with an 81% power, an ɑ level of 0.05, and a potential dropout rate of 20%, we found that a sample size in the intervention and control groups of 30 participants each was necessary to detect a clinically relevant difference of 3 QoL points between these groups [21]. Our study was conducted on all students in grades 4–5 in the primary schools and grades 7–8 in the middle schools over a single time period. Children in grades 6 and 9 were not participants in this study, as their matriculation to the next grade level would have caused them to miss follow-up visits necessary for this study. In total, 948 participants were recruited to participate in our study, including 479 boys and 469 girls, of whom 642 were primary school students and 306 were middle school students. The duration of intervention in this study was 1 year, with some students being lost to follow-up during the follow-up period. The study was approved by the Biomedical Ethics Committee of Peking University (IRB 00001052–13,034) and the ethical committee of Chongqing Medical University. Written informed consent was obtained from students and their parents before this study was conducted.

**Fig. 1:**
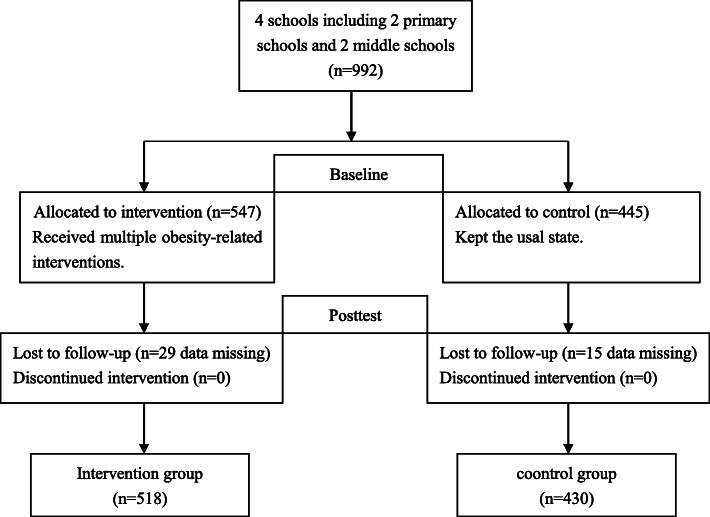
Flow chart of study

The baseline investigations were conducted in November 2016, and involved 445 students in the control group and 547 students in the intervention group that had completed the Adolescent Quality of Life Scale and provided baseline information. After the 1-year intervention period from November 2016 to November 2017, we conducted the final assessment in December 2017 with 430 students in the control group and 518 students in the intervention group returning to provide all of the same information collected at baseline. Figure 1 shows a flow chart of the study design.

Students filled out all questionnaires in a classroom setting with the help of their head teachers. Investigators, including 6 postgraduates and 2 teachers, were first trained by professors regarding the proper administration of this questionnaire before investigation. At the beginning of the investigation, these investigators informed all participants of the study aim, significance, and process, and obtained their informed consent. Next, the questionnaire instructions were read to students, who were then instructed to complete these forms in their entirety. All questionnaires were then collected from students and immediately reviewed, with any questionnaires that were missing or that contained mistakes being returned to the appropriate students for modification.

During the intervention period, the intervention group was supervised once per month. Through an interview form, we collected feedback regarding the intervention activities, problems arising during the intervention process, and suggestions and general opinions regarding the study, with improvements being made as appropriate. We also contacted monitors and head teachers to help supervise the diet and physical activity of these study subjects through these monthly check-ins. If subjects did not complete their intervention tasks as required, we contacted their parents and/or schools to ask them to complete the task.

### Family-individual-school-based comprehensive intervention

Table 1 details the family-individual-school-based comprehensive intervention measures employed in our study. This joint intervention strategy included family-, individual-, and school-based interventions involving three aspects: health education, diet, and physical activity which were targeted in accordance with previous surveys of obesity-related interventions [21, 23]. Teachers were the agents for the school-based interventions, while parents were the agents for the family-based interventions.

**Table 1:** Family-Individual-School-based comprehensive interventions for combating obesity

Intervention	Family	Individual	School
Health education	Providing “Happy Exercise, Healthy Diet [22]” proposals to parents (see additional file 1). Encouraging parental participation in obesity-related lectures.	Providing students with a book named “How to Avoid Obesity: Happy Exercise, Healthy Diet” [23] and lectures about reasonable diets and healthy exercise.	Health education courses once a month, and obesity-related health knowledge dissemination through posters, wall newspapers, radio, electronic screens, and websites.
Diet activity	Providing information to parents about reasonable diets, instructing parents about healthy dietary habits in children according to Chinese dietary guidelines (see additional file 2).	Encouraging students to consume at least 300-500 g of vegetables and 200-400 g of fruits per day, with a target meat intake of 80-110 g per day that can be adjusted based on energy consumption; not consume sugary drinks.	Providing purified water to students and prohibiting unhealthy snacks and sugary drinks from entering the classroom.
Physical activity	Providing information to parents about aerobic exercise; encouraging them to promote healthy exercise in their children (see additional file 3).	Encouraging students to control TV and computer usage for less than two hours per day and perform medium or high intensity exercise one hour per day.	Carrying out “Sunshine Sports Activities” through utilizing big break between classes and promising physical exercise one hour per day.

This comprehensive intervention approach involved informing participants of the risk factors and health consequences of being overweight or obese, while also introducing actionable prevention strategies and emphasizing that positive beliefs and health education offer the best means of avoiding these unhealthy behaviors, thus allowing these children to attain an improved QoL. We additionally provided these students with a healthy diet plan based on current Chinese dietary guidelines and provided a formulated exercise plan (Additional files 2-3).

### Measures

Body mass index (BMI) was determined for each participant based on their height and weight, as obtained from the Shapingba Primary and Secondary School Students Health Care Center in Chongqing. Height was accurate to within 0.1 cm, and weight was accurate to within 0.1 kg. BMI = weight (kg) / height (m^2^).

The Adolescent Quality of Life Scale [24] which includes physical(8 items), psychological(11 items), social(14 items), and pubertal dimensions(6 items) involving 10 factors (somatic symptoms, sleep status, negative emotions, aggressive emotions, school life, family life, peer relationships, appearance experiences, pubertal feelings, and pubertal cognition) was used to assess the QoL for study participants. Items regarding the frequencies of particular phenomena presented students with 5 options: “never (5 points)”,” almost never (4 points)”,” sometimes (3 points)”,” often (2 points)” and “always (1 point)”, with the exception of the item “How often do you take the initiative to understand puberty?” which included the following options: “never (1 point)”,” almost never (2 points)”,” sometimes (3 points)”,” often (4 points)” and “always (5 points)”. Items about life satisfaction also offered 5 options: “Very dissatisfied (1 point)”, “unsatisfactory (2 points)”, “neutral (3 points)”, “satisfactory (4 points)” and “very satisfactory (5 points)”. We tested the reliability and validity of this Adolescent Quality of Life Scale in primary and middle school students in Chongqing, with the resultant ɑ coefficients of the physical, social, psychological, and pubertal dimensions, and the overall scale being 0.81, 0.77, 0.85, 0.64, and 0.89, respectively. Retest reliability values were also calculated as being 0.76, 0.78, 0.82, 0.72, and 0.88, respectively [24]. These previous results suggest that this scale is a reliable means of accurately assessing adolescent QoL.

We collected the key baseline demographic information with the potential to impact QoL from each participant, including school, sex, grade, class, family economic status, family relationships, parental educational level, parenting style, academic pressure, academic records, number of close friends, and similar factors through a baseline informational questionnaire. Family economic status, family relationships, and academic pressure were determined by subjective self-judgments relative to each participants’ peers. Parenting style options included democratic (respectful, understanding, and supportive), autocratic (strict, with excessive behavioral regulations), indulgent, and neglectful.

### Data analysis

SPSS 21.0 and EpiData 3.1 were used to analyze the data from this study. We initially tested the equilibrium between the control and intervention groups via Chi-squared tests and t-tests. Then, paired t-tests were utilized to test changes in participant QoL after intervention in the control and intervention groups. We then calculated the changes in QoL over baseline and used this variable along with other assessed items in a multi-level mixed effect analysis with school level as a random effect in order to explore the effectiveness of the interventions on particular aspects of QoL, and to assess group-specific differences in outcomes (boys vs. girls). This approach allows us to control for the influence of different schools and potential confounding factors (sex, degree of education, only child status, family economic situation, family relationships, educational level of parents, parenting style, study record, study pressure, and number of close friends) on study outcomes.

## Results

### Distribution of sociodemographic characteristics between control group and intervention group

The average age of participants in this study was 11.39 ± 1.81 years (range: 9–17). Table 2 demonstrates that there were no significant differences in terms of sex, degree of education, family economic status, family relationships, parental educational levels, parenting style, academic records, academic pressure, or number of close friends (*P* > 0.05) at baseline between the control and intervention groups. Only child status (*P* = 0.030) and BMI (*P* = 0.001) did differ significantly between these groups at baseline. Therefore, when comparing the differences in QoL between the intervention group and the control group, we controlled for the impacts of only child status and baseline BMI on QoL via a multiple linear regression analysis approach.

**Table 2:** Equilibrium test of socio-demographic characteristics between intervention and control groups in baseline(*n* = 1256)

Socio-demographic characteristics		Control	Intervention	χ^2^/t	P
Sex	Boy	214	265	0.182	0.670
	Girl	216	253		
Educational degree	Primary school	289	353	0.094	0.759
	Middle school	141	165		
Only child	Yes	196	200	4.695	0.030
	No	234	318		
Family economic status	Poor	43	63	1.752	0.416
	Medium	240	291		
	Good	110	118		
Family relationships	Not harmonious	20	24	3.824	0.148
	Medium	157	221		
	Harmonious	253	273		
Educational level of father	Middle school or lower	215	263	0.190	0.910
	High/technical secondary school	157	190		
	Junior college or higher	58	65		
Educational level of mother	Middle school or lower	211	287	5.264	0.072
	High/technical secondary school	166	186		
	Junior college or higher	53	45		
Parenting style	Democracy	233	276	3.464	0.325
	Autarchy	138	187		
	Indulgence	50	44		
	Neglect	9	11		
Academic records	Bad	130	153	0.485	0.785
	Medium	163	189		
	Good	137	176		
Academic pressure	Low	74	93	4.313	0.116
	Medium	197	266		
	Great	159	159		
Number of close friends	≤ 2	114	148	0.668	0.716
	3–5	157	178		
	≥ 6	159	192		
	BMI at baseline	18.52 ± 3.22	17.83 ± 3.02	3.194	0.001

### Comparison of baseline and post-intervention QoL between control and intervention groups

Table 3 shows the QoL at baseline and after intervention in both the control and intervention groups. There were significant improvements in the psychological, social, and pubertal dimensions of the QoL scale as well as in total QoL (*P* < 0.05), but not in the physical dimension (*p* > 0.05), in both the control and intervention groups. Psychological, social, and pubertal dimensions, as well as overall QoL, increased by 2.77, 2.20, 1.95, and 6.86 points, respectively, following the intervention period in the intervention group. In the control group, these parameters were also increased at follow-up relative to baseline, though the increase was not as substantial as for the intervention group.

**Table 3:** Comparison of QoL pretest and posttest between two groups. (Paired t-test)

Group	QoL	Baseline	After intervention	Difference	t	P
		M ± SD	M ± SD			
Control	Physical	30.40 ± 5.17	30.34 ± 5.41	−0.06	−0.321	0.818
	Psychological	40.12 ± 6.81	40.90 ± 7.25	0.79	2.082	0.038
	Social	50.77 ± 8.41	52.53 ± 8.76	1.76	4.238	0.000
	Pubertal	20.28 ± 3.68	21.43 ± 3.28	1.15	5.635	0.000
	Total QoL	141.57 ± 17.98	145.20 ± 19.56	3.64	4.150	0.000
Intervention	Physical	30.07 ± 5.19	30.01 ± 5.33	−0.07	−0.282	0.778
	Psychological	38.66 ± 7.25	41.44 ± 7.33	2.77	8.115	0.000
	Social	50.99 ± 8.19	53.19 ± 8.88	2.20	5.872	0.000
	Pubertal	19.54 ± 4.10	21.49 ± 3.66	1.95	9.501	0.000
	Total QoL	139.27 ± 18.37	146.13 ± 20.08	6.86	8.405	0.000

Note: Difference = After invention-Baseline

### Comparison of the QoL in the control and intervention groups

A multi-level mixed effect analysis was next conducted using a line model with school level as a random effect. Table 4 details the B coefficient value, SE, t, and *P*-values for the effect of the intervention on changes in QoL based on a multiple linear regression analysis controlling for potential confounding variables with the control group as a reference. Significant differences were identified in the psychological (B = 1.883, SE = 0.646, *P* = 0.004) and pubertal (B = 0.853, SE = 0.296, *P* = 0.004) dimensions, as well as in overall QoL (B = 3.024, SE = 1.214, *P* = 0.013), but not in physical or social dimensions (*P* > 0.05).

Interventional efficacy varied in different subgroups (Table 4). There were significant differences in psychological (B = 2.605, SE = 0.752, *P* = 0.001) and pubertal (B = 0.864, SE = 0.417, *P* = 0.039) dimension scores and in overall QoL (B = 4.904, SE = 1.714, *P* = 0.004) among boys, with no significant differences in physical or social dimensions (*P* > 0.05). In contrast, no significant differences were detected for any dimension among girls (*P* > 0.05).

**Table 4:** Multi-level mixed effect analysis for intervention associated with QoL (subject: schools)

Group	Dependent	B	SE	t	P
All subjects	Physical	−0.072	0.377	−0.192	0.848
	Psychological	1.883	0.646	2.917	**0.004**
	Social	0.221	0.702	0.314	0.754
	Pubertal	0.853	0.296	2.881	**0.004**
	Total QoL	3.024	1.214	2.491	**0.013**
Boy	Physical	0.654	0.955	0.648	0.494
	Psychological	2.605	0.752	3.464	**0.001**
	Social	1.006	0.792	1.271	0.205
	Pubertal	0.864	0.417	2.070	**0.039**
	Total QoL	4.904	1.714	2.862	**0.004**
Girl	Physical	−0.687	0.482	−1.426	0.154
	Psychological	1.097	0.728	1.507	0.133
	Social	−0.494	0.821	−0.602	0.548
	Pubertal	0.478	0.660	0.723	0.470
	Total QoL	0.519	1.772	0.293	0.770

Note: Independent variable: Control versus Intervention

Dependent variable: the difference of QoL between baseline and after intervention

Other corresponding confounding factors in model: BMI at baseline, sex (no in boy and girl), education degree, only child, family economy, family relationship, educational level of parents, parenting style, study record, academic pressure and number of good friends

## Discussion

QoL is defined by the WHO as an individual’s perception of their position in life in the context of the culture and value systems in which they live and in relation to their goals, expectations, standards, and concerns [25]. The QoL index is multidimensional and artificial construct [26], and refers not only to the absence of disease or infirmity but also to a state of complete physical, mental, and social well-being [27]. Previous studies on QoL have mainly focused on its physical, psychological, and social dimensions [28]. However, as participants in our study were in adolescence, the impact of pubertal development on their QoL [29] was also assessed by incorporating a pubertal dimension into our assessment tool that assessed puberty-related feelings and pubertal cognition. This dimension, along with the standard physical, social, and psychological dimensions, was used to explore the efficacy of family-individual-school-based interventions on QoL in school-aged children and adolescents.

In our study, we found that the psychological, social, and pubertal dimensions as well as overall QoL were all significantly improved following the implementation of our family-individual-school-based intervention strategy. QoL was also increased at final follow-up in the control group, although the improvement in the intervention group was greater. Our results revealed that the improvements in psychological QoL were greatest in the intervention group, increasing by 2.77 following intervention. Increases in the pubertal dimension were less profound, while overall QoL was substantially improved by 6.86 points following intervention.

We observed superior enhancement in the psychological QoL of students in the intervention group, consistent with the work of Kovacs E et al. [30], although inconsistent with the work of Warkentin LM et al. [31], who found that physical activity did not significantly improve psychological QoL. At present in China, many thin children, and particularly girls, often report feeling somewhat fat [32], indicating a lack of body image confidence in these children. As our intervention included obesity-related health education, it encouraged participants to accept their own appearance and body image in a positive light, which may account for some of the observed improvements in the psychological QoL dimension scores. There is increasing evidence indicating that physical activity is positively associated with self-esteem and self-efficacy [33, 34], and these favorable traits can improve confidence and life satisfaction, thus contributing to higher QoL. With respect to the pubertal dimension of our QoL scale, the positive changes observed in the intervention group were also greater than those in the control group. No previous studies have focused on this dimension, and so we are unable to compare these results with those of other studies. All participants in this study were adolescents, and as such were undergoing major physical and psychological changes, with psychological maturation often occurring later than physical maturation during this key period. Being overweight or obese will often result in the earlier onset of puberty, widening this gap further [35]. For instance, girls with more body fat content are more likely to experience menarche at younger ages [36]. As our comprehensive obesity-related strategy can reduce fat mass in children and adolescents, it can narrow this gap and thereby improve pubertal QoL.

However, our research identified no intervention-related impact on the physical QoL dimension relative to the control group, which was inconsistent with previous results suggesting that physical exercise can improve QoL [31, 37, 38]. This inconsistency is likely a result of the particular study populations used in each study, as a previous literature review found the influence of weight status on QoL was greater in clinical populations than in normal populations [28]. Our participants were all children in grades 4–5 and 7–9, and all had good physical QoL, so there was little room for improvement of this dimension, whereas in previous studies regarding the effect of obesity-related interventions on QoL [6, 7, 11], participants were largely obese or overweight individuals that had a lower and less stable physical QoL. Therefore, it is more difficult to improve physical QoL for normal participants than for those individuals with physical health issues. We also found that our intervention measures did not affect social QoL, unlike the results of a previous study by Uritani D et al. [39]. This difference may be a consequence of the fact that children and adolescents become more self-centered and rebellious during adolescence but lack sufficient competence to deal with it, leading to more conflicts between parents and adolescents, conflicts between peers, and conflicts between teachers and students. Therefore, in future studies, it will be important to further examine adolescent mental health.

Our study results suggested that the efficacy of this intervention strategy varied between boys and girls, with a much clearer effect in boys, which is inconsistent with the results of previous studies [40, 41]. Brown DW et al. [42] have previously found that physical activity improved quality of life for both men and women. The sex difference observed in our study may be due to the fact that the effect of physical exercise on overall QoL is better than that of diet control [43]. Boys prefer physical exercise and were more likely to adhere to the intervention measures in the present study, and Yiyi Ouyang et al. found that males were more likely to have greater self-efficacy and self-affirmation relative to females, potentially further explaining this sex difference [44]. In addition, there are some prior studies indicating that boys suffer greater QoL impairment than do girls when obese or overweight [45], making it easier to improve the QoL of boys relative to that of girls. However, these results still raise questions regarding pre-adolescent emotional development, puberty issues, and self-image from a gender perspective and make it uncertain as to whether this approach can be generalized to other populations. Therefore, these disparities between boys and girls require further in-depth research.

## Conclusion

Family-individual-school-based interventions incorporating obesity-related health education, physical exercise, and diet control can improve psychological and pubertal well-being, as well as overall QoL in children, particularly among boys.

### Limitations

There are several limitations to the current study. While the obesity-related intervention in this study was a comprehensive measure involving school-, family- and individual-based obesity-related health education, physical exercise, and diet control, we did not explore the significance of these three interventions separately. As such, we are unable to determine which, if any, of these interventions is most efficacious. We also did not systematically assess or record subject intervention activities during the study period, and as such only monitors and head teachers conducted quality control for this study, potentially reducing the intervention effect.

## Supplementary information

**Additional file 1.**

**Additional file 2.**

**Additional file 3.**

## Data Availability

Not applicable.

## Abbreviations

QoL: Quality of life
RCT: Randomized controlled trial
BMI: Body mass index
WHO: World Health Organization
