# Supplementary material for: The impacts of multiple obesity-related interventions on quality of life in children and adolescents: a randomized controlled trial
Source: Health Qual Life Outcomes. 2020 Jul 6;18:213. doi: 10.1186/s12955-020-01459-0 (PMC7336614; doi:10.1186/s12955-020-01459-0)
Supplement: Supplementary file 1 — Additional file 1. [file 12955_2020_1459_MOESM1_ESM.doc]

**“Happy Exercise, Healthy Diet” proposals:**

1. Food is diverse, cereals are dominant.
2. Keep the balance between diet and exercise.
3. Eat more fruits, vegetables, milk and soy.
4. Eat fish, poultry, eggs, and lean meat in moderation.
5. Less salt, less oil and sugar control in cooking food, and no drinking for adolescents.
6. Eliminate waste and developing good habits.
