# Supplementary material for: The impacts of multiple obesity-related interventions on quality of life in children and adolescents: a randomized controlled trial
Source: Health Qual Life Outcomes. 2020 Jul 6;18:213. doi: 10.1186/s12955-020-01459-0 (PMC7336614; doi:10.1186/s12955-020-01459-0)
Supplement: Supplementary file 2 — Additional file 2. [file 12955_2020_1459_MOESM2_ESM.doc]

**Specific reasonable diet:**

1. The daily diet should include cereals, vegetables, fruits, livestock, fish, eggs, soy nuts and other foods. Eat more than 12 kinds of foods per day, more than 25 kinds per week. Every day, 250g-400g of potato food is taken, of which 50g-150g of whole grains and miscellaneous beans, and 50g-100g of potatoes.
2. Vegetables and fruits are an important part of a balanced diet. Milk is rich in calcium and high quality protein. Every meals have vegetables. Ensure that 300 to 500 grams of vegetables are ingested per day, and dark vegetables should account for 1/2. Eat fruit every day. Guaranteed to take 200 to 350 grams of fresh fruit every day, juice can not replace fresh fruit. Eat a variety of dairy products which is Equivalent to 300 grams of liquid milk per day. Regularly eat soy products and eat nuts in moderation.
3. Fish, poultry, eggs and lean meat should be taken in moderation. Eat 280-525 grams of fish, 280-525 grams of livestock and poultry meat and 280-350 grams of eggs per week . The average total intake is 120-200 grams per day. Priority is given to fish and poultry. Eat eggs and not leave the egg yolk. Eat less fat, smoked and cured meat products.
4. Develop light habits and eat less high-salt and fried foods. The salt is not more than 6 grams per day, and the cooking oil is 25-30 grams per day. Control the intake of added sugar, and take no more than 50 grams per day, preferably under 25 grams. Drink plenty of water, 6-7 cups per day (at least 2000 ml), and adjust the amount of water according to exercise. Promote drinking boiled water and tea without drinking or drinking sugary drinks. Children and teenagers should not drink alcohol.
5. Appreciate the food , prepare meals on demand and promote no waste of meals. Choose fresh and healthy food and the right way to cook. When preparing and cooking food, the raw food and the cooked food should be separated, and the cooked food should be completely heated when it is heated twice. Learn to read food labels and choose foods reasonably. Go home to eat, enjoy food and family.
