# Supplementary material for: The impacts of multiple obesity-related interventions on quality of life in children and adolescents: a randomized controlled trial
Source: Health Qual Life Outcomes. 2020 Jul 6;18:213. doi: 10.1186/s12955-020-01459-0 (PMC7336614; doi:10.1186/s12955-020-01459-0)
Supplement: Supplementary file 3 — Additional file 3. [file 12955_2020_1459_MOESM3_ESM.doc]

**Specific happy exercise:**

1. Maintain daily exercise and healthy weight. You can't eat too much, control the total energy intake, and maintain energy balance. Adhere to daily physical activity. Perform moderate-intensity physical activity at least 5 days a week for more than 150 minutes and at least 3 hours high-intensity physical activities per week.
2. Active physical activity is best taken 6,000 steps per day. Reduce sedentary time and stand up to exercise for a while every hour.
3. Reduce the static activity time: leave the seat to do physical activities such as resting or playing game for 10 minutes between classes; when doing homework outside the classroom, we should do stretching exercises one time every 40 minutes; schedules are kept regular on weekends and holiday
4. Try to do other physical exercises: walk as much as possible to school every day. If the home is far from the school, we can get off at one stop in advance and walk to school. Walk up and down the stairs every day, and do not take the elevator. Actively do some housework as much as you can, such as washing dishes, wiping tables, etc.
5. Sports classification:

Mild: stretching exercises; walking; some housework activities such as watering flowers, wiping tables, sweeping the floor, etc.

Medium intensity: jogging; dancing; aerobics; going up and down the stairs; playing table tennis; swimming, etc.

High intensity: playing basketball; playing football; running; jumping rope; climbing; carrying things weighing more than 11 kilograms, etc.
